# Rho-Kinase Activation in Leukocytes Plays a Pivotal Role in Myocardial Ischemia/Reperfusion Injury

**DOI:** 10.1371/journal.pone.0092242

**Published:** 2014-03-17

**Authors:** Katsunori Kitano, Soichiro Usui, Hiroshi Ootsuji, Shin-ichiro Takashima, Daisuke Kobayashi, Hisayoshi Murai, Hiroshi Furusho, Ayano Nomura, Shuichi Kaneko, Masayuki Takamura

**Affiliations:** Department of Disease Control and Homeostasis, Kanazawa University Graduate School of Medical Science, Kanazawa, Ishikawa, Japan; Rutgers New Jersey Medical School, United States of America

## Abstract

The Rho/Rho-kinase pathway plays an important role in many cardiovascular diseases such as hypertension, atherosclerosis, heart failure, and myocardial infarction. Although previous studies have shown that Rho-kinase inhibitors reduce ischemia/reperfusion (I/R) injury and cytokine production, the role of Rho-kinase in leukocytes during I/R injury is not well understood. Mice were subjected to 30-min ischemia and reperfusion. Rho-kinase activity was significantly greater in leukocytes subjected to myocardial I/R compared to the sham-operated mice. Administration of fasudil, a Rho-kinase inhibitor, significantly reduced the I/R-induced expression of the proinflammatory cytokines interleukin (IL)-6, C-C motif chemoattractant ligand 2 (CCL2), and tumor necrosis factor (TNF)-α, in leukocytes, compared with saline as the vehicle. Furthermore, fasudil decreased I/R-induced myocardial infarction/area at risk (IA) and I/R-induced leukocyte infiltration in the myocardium. Interestingly, IA in fasudil-administered mice with leukocyte depletion was similar to that in fasudil-administered mice. I/R also resulted in remarkable increases in the mRNA expression levels of the proinflammatory cytokines TNF-α, IL-6, and CCL2 in the heart. Inhibition of Rho-kinase activation in leukocytes has an important role in fasudil-induced cardioprotective effects. Hence, inhibition of Rho-kinase may be an additional therapeutic intervention for the treatment of acute coronary syndrome.

## Introduction

Despite improvements in treatments after acute coronary syndrome (ACS), patients are still at risk of developing significant myocardial necrosis/apoptosis and remodeling [1]. Reperfusion of ischemic myocardium is an essential strategy for salvaging tissue from inevitable death. However, the early opening of an occluded coronary artery sometimes induces myocardial ischemia/reperfusion (I/R) injury [2], characterized by a cascade of acutely initiated local inflammatory responses, metabolic disorder, cell death, and subsequent cardiac dysfunction and remodeling. Increasing evidence suggests that multiple factors are involved in I/R injury, such as Ca^++^ overload, generation of oxidative stress, cytokine production, and neutrophil infiltration [3].

The transmission of extracellular stress signals such as I/R injury into an intracellular response have been shown to involve small guanosine-5′-triphosphate-binding proteins such as those of the Rho family. Rho-kinase, a serine/threonine kinase, has been identified as a downstream effector of Rho. The Rho/Rho-kinase axis plays an important role in cardiovascular diseases such as hypertension, heart failure, myocardial infarction, and atherosclerosis [4]–[6]. Fasudil, a Rho-kinase inhibitor, has a beneficial effect in the treatment of acute ischemic stroke and cerebral vasospasm [7]. The efficacy of fasudil is related to a potent vasodilator effect and inhibition of neutrophil infiltration. Stimulation of Rho-kinase has been implicated in infarct development after myocardial I/R through the mechanism of reduced eNOS activity via the phosphatidyl inositol 3-kinase/Akt pathway [8], [9] in the heart.

Neutrophil activation also contributes to I/R injury by obstructing capillary vessels and releasing vasospastic substrates and inflammatory cytokines [10]. Neutrophils release huge amounts of cytokines during myocardial I/R [11], and neutrophil inhibition with anti-polymorphonuclear antibody as well as neutrophil depletion reduced I/R-induced infarct size [12] and the production of reactive oxygen species and inflammatory cytokines [13]. Rho-kinase inhibitors also reduced I/R-induced myocardial infarction and cytokine production in mice models [14]. In the clinical settings, Rho-kinase activity in peripheral blood leukocytes tended to be higher in coronary artery disease subjects compared with healthy individuals [15]. It is not clear whether the suppression of Rho-kinase activity in leukocytes contributes to reduce productions of inflammatory cytokines and myocardial damage following I/R. Therefore, the aim of the present study was to clarify whether the Rho/Rho-kinase axis in leukocytes contributes to reduce myocardial I/R injury.

## Materials and Methods

### Ethics statement

All animal protocol was performed according to the Guide for the Care and Use of Laboratory Animals in Kanazawa University, which strictly conforms to the *Guide for the Care and Use of Laboratory Animals*, published by the US National Institutes of Health (NIH, Bethesda, MD). The protocol was approved by the ethical committee of Kanazawa University (Approval NO. 132814).

### Ischemia/reperfusion

Male C57/B6J mice (Charles River Laboratories, Yokohama, Japan) were intraperitoneally administered either vehicle (NaCl 0.9%) or the Rho-kinase inhibitor fasudil (10 mg/kg; Asahi Kasei Pharma Corporation, Tokyo, Japan) 1 h before the operation. The mice were subjected to I/R or sham operation as described elsewhere [16]. Briefly, a lateral thoracotomy was performed in the anesthetized and ventilated mice, and a 7-0 suture was looped under the left descending coronary artery for the induction of a coronary artery occlusion for 30 min, followed by 24 h of reperfusion. The hearts were then removed, and the area at risk (AAR) and infarct size (IA) were determined using staining with Evans blue (EB) and 2,3,5-triphenyltetrazolium chloride (TTC). The EB-stained areas (blue staining) indicate non-ischemic regions; the TTC-stained areas (red staining) indicate ischemic but viable tissue; the EB and TTC negatively stained areas (white) indicate myocardial infarct.

### Evaluation of apoptosis in tissue sections

DNA fragmentation was detected *in situ* using terminal deoxynucleotidyl transferase dUTP nick-end labeling (TUNEL) [17]. Briefly, deparaffinized sections were incubated with proteinase K and DNA fragments were labeled with fluorescein-conjugated dUTP using TdT (Roche Molecular Biochemicals, Mannheim, Germany). Nuclear density was determined by manual counting of 4′-6-diamidino-2-phenylindole (DAPI)-stained nuclei in 10 fields for each animal using the 40× objective, and the number of TUNEL-positive nuclei was counted by examination of the entire section, using the same power objective.

### Cell isolation and fluorescence-activated cell sorting (FACS) analysis

We evaluated infiltrating cells using FACS analysis. To obtain single cell suspensions, hearts were perfused with phosphate-buffered saline (PBS), isolated, and incubated in collagenase type II (Worthington Laboratories, Lakewood, NJ) enzyme solution for 30 min at 37°C with gentle agitation [18]. The cells were filtered with a 40-mm cell strainer, and washed with MACS buffer (PBS, 0.5% BSA, 2 mM EDTA, degassed) twice. Subsequently, the cells were incubated with fluorescein isothiocyanate (FITC)-conjugated anti-mouse CD45 and PE-conjugated anti-mouse Gr-1 antibodies for 20 min on ice. Cells were analyzed on a FACSCalibur instrument (BD Biosciences, San Jose, CA). Data were analyzed using FlowJo software (TreeStar, Ashland, OR).

### Western blot analysis

Leukocytes were isolated according to the method previously described with slight modifications [19]. Mouse whole blood was collected into Hank's balanced saline solution (HBSS)-EDTA. After centrifugation (400×*g*, 10 min, 4°C), cells were resuspended in 1 ml HBSS-EDTA. The cells were laid on with a three-layer Percoll® gradient of 78%, 69%, and 52% Percoll (Amersham Pharmacia Biotech, Piscataway, NJ), respectively, and centrifuged (1500×*g*, 30 min, room temperature) without braking. The leukocytes were collected from the 69%/78% interface and the upper part of the 78% layer. The remaining red cells were eliminated by hypotonic lysis. After one wash and centrifugation, leukocytes were homogenized on ice with the RIPA buffer. The lysates were centrifuged at 15,000×*g* for 5 min. Equal amounts of proteins were loaded, separated by electrophoresis on sodium dodecyl sulfate-polyacrylamide gels, and transferred onto a polyvinylidene fluoride membrane. The antibodies used included anti-phospho-MYPT and MYPT (Cell Signaling Technology, Danvers, MA). The band density was quantified using Image J software (NIH).

### Isolation of mRNA and quantitative real-time polymerase chain reaction

Total RNA was isolated using an RNeasy Fibrous Tissue Kit (Qiagen, Valencia, CA) for the heart tissue and a QIAamp RNA Blood Kit for the leukocytes (Qiagen) according to the manufacturer's protocols[20]. Total RNA (100 ng) was used to generate cDNA using the TaqMan Universal Master Mix (Applied Biosystems, Foster City, CA), according to the manufacturer's protocol. Quantitative real-time polymerase chain reaction (qRT-PCR) analysis was performed using the ABI Prism 7300 sequence detection system (Applied Biosystems). The following primers and TaqMan probes (Applied Biosystems) were used: ROCK1 (Mm00485745_m1), ROCK2 (Mm01270843_m1), TNF-α (Mm00443258_m1), interleukin (IL)-6 (Il6, Mm99999064_m1), C-C motif ligand 2 (CCL2, ID no. Mm00441242_m1), and IL-10 (Il10, ID no. Mm01288386_m1). Glyceraldehyde-3-phosphate dehydrogenase (GAPDH; ID no. Mm99999915_g1) was used as an endogenous control.

### Measurement of myeloperoxidase activity

Ischemic left ventricular (LV) tissue samples were homogenized in 20 mM potassium phosphate and then centrifuged at 20,000×*g* for 30 min [21]. The pellets were then frozen for 12 h. After thawing, the pellets were added to a solution and measured using enzyme-linked immunosorbent assay (ELISA) according to the manufacturer's recommended protocols (Cayman Chemical Company, Ann Arbor, MI).

### ELISA for TNF-α, IL-6, CCL2, and IL-10

We monitored TNF-α, IL-6, CCL2, and IL-10 levels in serum and cell culture supernates using a mouse TNF-α, IL-6, CCL2, and IL-10 Quantikine ELISA Kit (R&D Systems) according to the manufacturer's protocols.

### Leukocyte chemotaxis assay

A leukocyte chemotasis assay was performed using a CytoSelect 96-well Migration Assay with 3-μm pores (Cell Biolabs, San Diego, CA) [22]. The assay was conducted following the manufacturer's protocols. Inducers of chemotaxis were added to the bottom wells in RPMI 1640 medium with 5% fetal calf serum (FCS). Leukocytes suspended in RPMI 1640 medium (5×10^4^ cells/50μl) were added to the top wells and incubated for 2 h. The data represent fold changes of transmigrated leukocytes compared to the wells filled with untreated control leukocytes.

### Histological analyses

Histological analyses of the heart sections were conducted as described previously [23]. The heart specimens were fixed with 10% neutral buffered formalin, embedded in paraffin, and sectioned at 6-μm thickness. For immunofluorescent staining, leukocytes were stained with anti-Gr-1 antibody (BD Biosciences). Alexa 488- and Alexa 594-conjugated secondary antibodies (Life Technologies Japan, Tokyo, Japan) were used. Nuclei were stained with DAPI.

### Neutrophils depletion

Neutrophil depletion was performed using adsorbed rabbit anti-mouse polymorphonuclear cell (PMN) serum (Accurate Chemicals, Westbury, NY) as described previously[24]. The anti-PMN serum was diluted 1∶10 in sterile saline, and mice were injected with 300μl every 3 days prior to I/R injury. Treatments with anti-PMN led to a 72% reduction in the average level of circulating neutrophils (p<0.01).

### Statistics

All values are expressed as mean ± SEM. Statistical analyses performed by either t test or one-way ANOVA followed by a post hoc Bonferroni-Dunn's comparison test. A value of P<0.05 was considered significant. Statistical analyses were conducted using GraphPad Prism (GraphPad Software, La Jolla, CA).

## Results

### Rho-kinase pathway is activated in leukocytes after ischemia reperfusion

**Figure 1:**
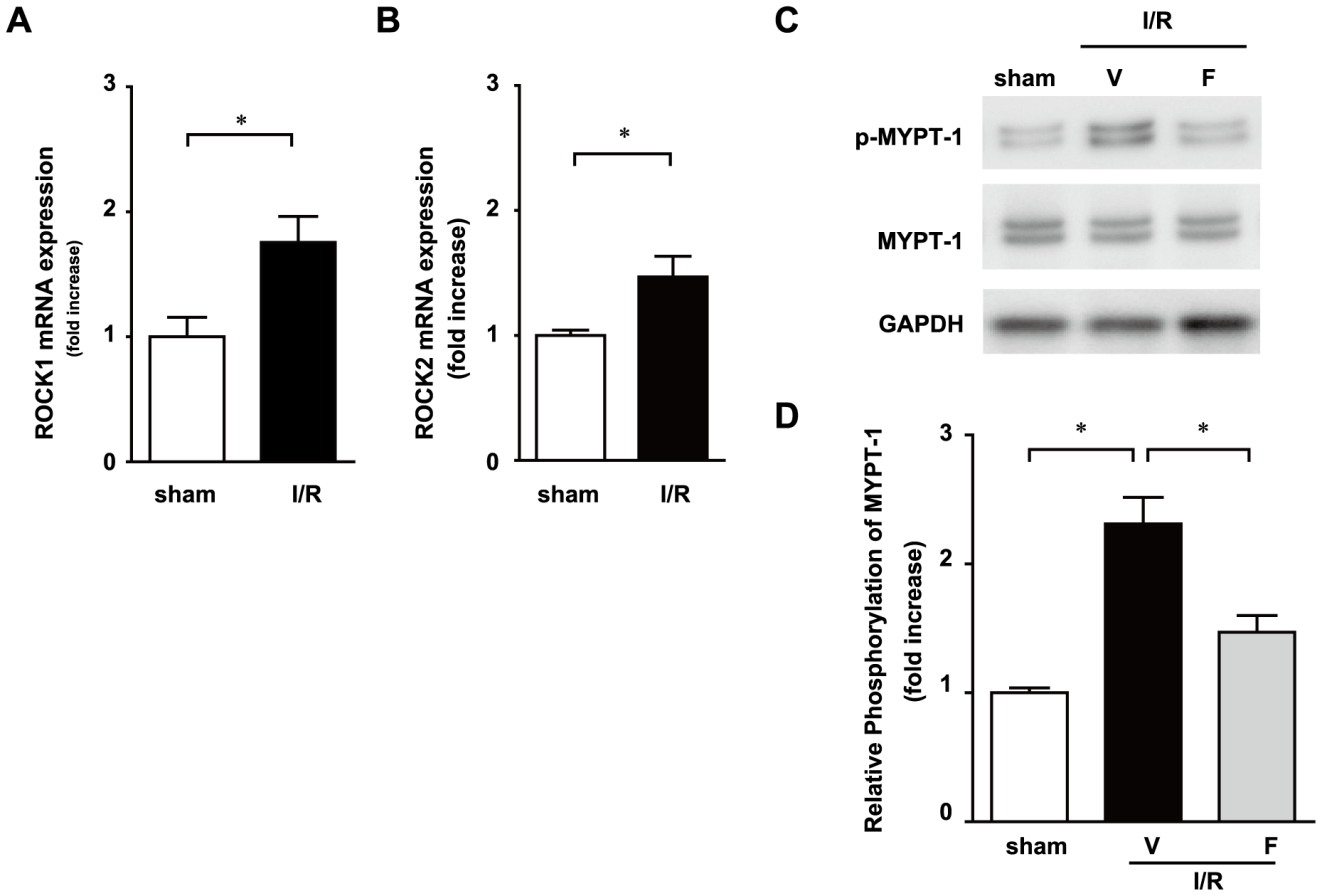
Rho kinase is activated in peripheral blood cells after 30-min ischemia and 12-h reperfusion (I/R). The mRNA expression of Rho kinase 1 (A) and 2 (B) in leukocytes 12 h after I/R was determined by quantitative RT-PCR. Expression levels of MYPT-1 and phosphorylated MYPT-1 were determined by immunoblotting (C). Results of the quantitative analysis of phosphorylated MYPT-1 are shown (D). Data represent means from at least 5 mice each. * p<0.05, ** p<0.01.

We examined the impact of I/R injury on the Rho-kinase pathway in leukocytes. Mice were subjected to 30 min of ischemia and a subsequent 12 h of reperfusion. The expression levels of Rho-associated, coiled-coil containing protein (ROCK)1 and ROCK2 mRNA were upregulated (Figure 1A and B) and the phosphorylation of myosin phosphatase-targeting subunit 1(MYPT-1), a downstream target of Rho-kinase, was increased in leukocytes obtained from I/R mice (Figure 1C and D). The phosphorylation levels of MYPT-1 were significantly less in the I/R with fasudil (I/R+F) group, the Rho-kinase inhibitor, than in the I/R with vehicle (I/R+V) group. These results suggest that the Rho/Rho-kinase pathway is activated in leukocytes following I/R.

### Inhibition of Rho-kinase activity reduces I/R-induced serum cytokine concentrations and expression levels in leukocytes

**Figure 2:**
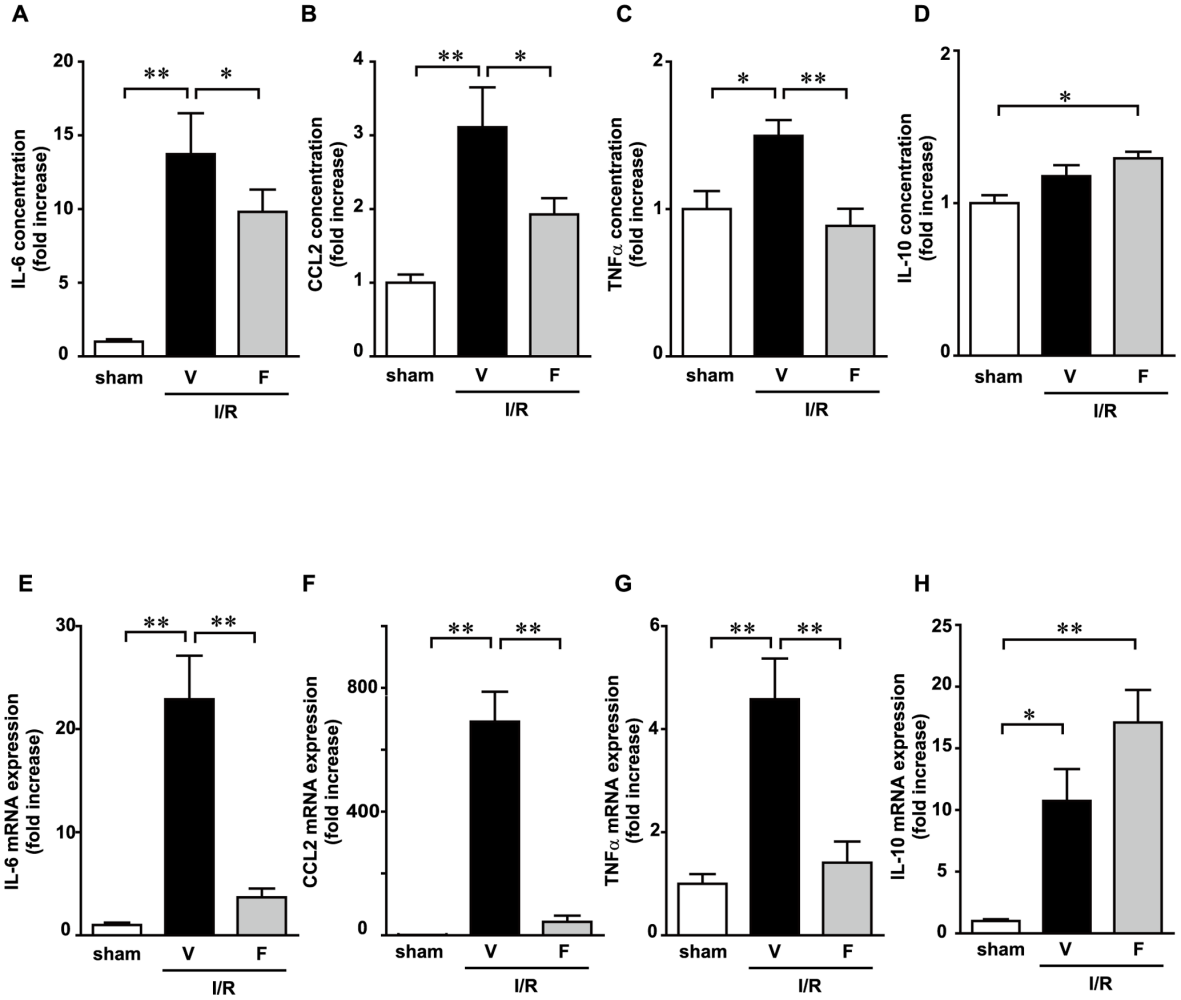
Fasudil reduces the production of various inflammatory cytokines. Serum samples were detected using an enzyme-linked immunosorbent assay at 12 h after ischemia/reperfusion (I/R). Interleukin (IL)-6 (A), chemokine (C-C motif) ligand 2 (CCL2) (B), tumor necrosis factor (TNF)-α (C) and IL-10 (D). Fasudil also reduces the ischemia/reperfusion (I/R)-induced expression of proinflammatory cytokines in leukocytes. Total RNA of leukocytes was collected at 12 h after I/R and detected using real-time polymerase chain reaction. The mRNA expression levels of IL-6 (E), CCL2 (F), TNF-α (G), and IL-10 (H) were assessed. Data represent means from at least 5 mice each. * p<0.05, ** p<0.01.

We measured cytokine levels in the serum 12 h after I/R. The serum concentrations of IL-6, CCL2 and TNF-α were significantly elevated in the I/R+V group compared with those in sham-operated mice (Figure 2 A, B and C), whereas the concentration of IL-10 was not significantly different between the 2 groups (Figure 2D). Increases in IL-6, CCL2, and TNF-α expression levels were significantly suppressed in the I/R+F group compared with those in the I/R+V group. It is interesting that the anti-inflammatory cytokine IL-10 was upregulated in the I/R+F group. Leukocytes are an important source of cytokines; therefore, we measured the mRNA expression of these cytokines in leukocytes. The expression levels of IL-6, CCL2, TNF-α and IL-10 were significantly induced in the I/R+V group compared with those in sham-operated mice (Figure 2, E, F, G and H). I/R-induced increases of proinflammatory cytokines, including IL-6, CCL2 and TNF-α, were suppressed by fasudil administration, whereas the anti-inflammatory cytokine IL-10 was upregulated by fasudil administration. These results suggest that Rho-kinase activation in leukocytes partially influences the production of cytokines and modulates the balance of inflammation during I/R.

### Inhibition of Rho-kinase activity reduces leukocyte infiltration after I/R in the heart

**Figure 3:**
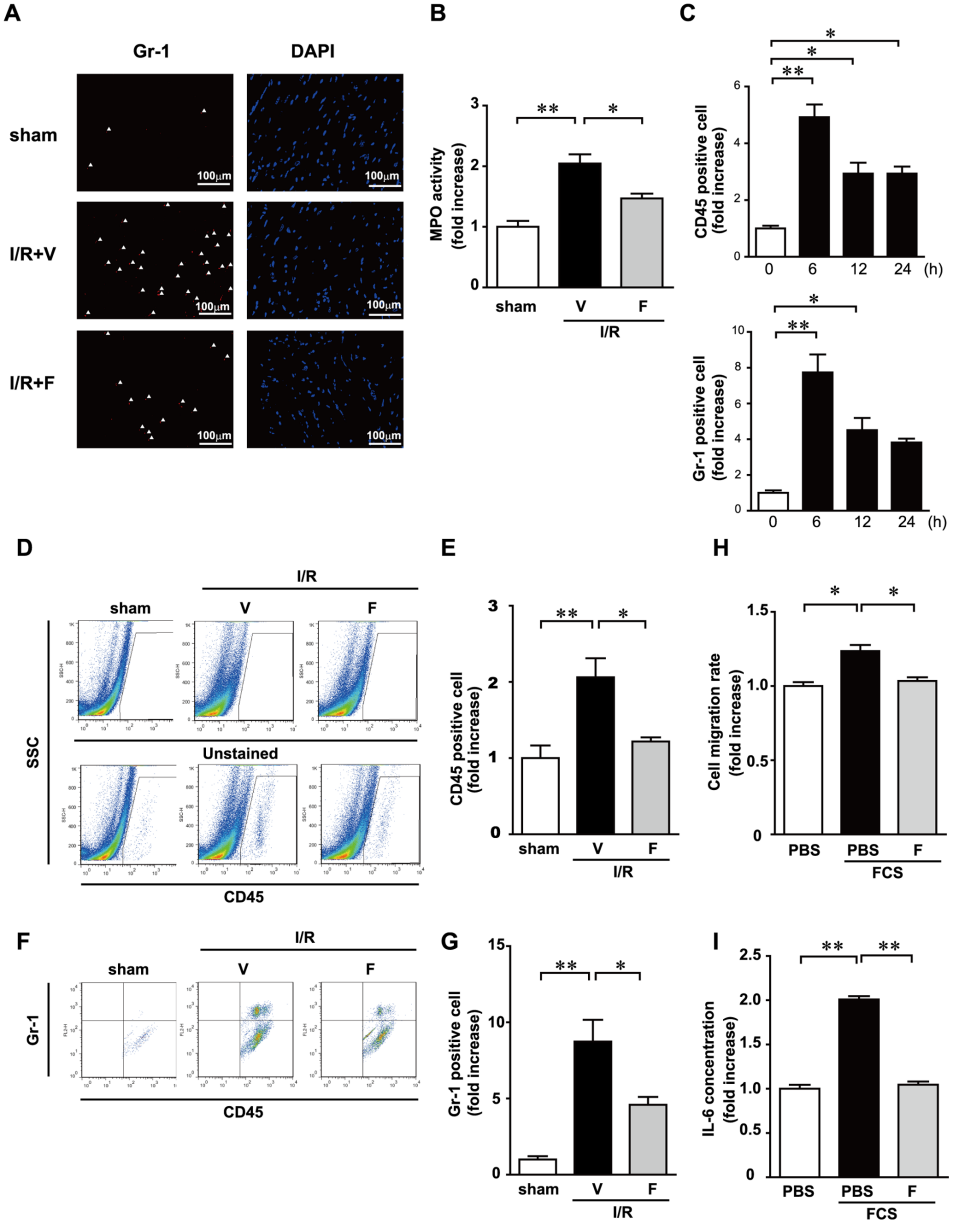
Fasudil reduces neutrophil infiltration after I/R. Immunohistochemical analysis of neutrophils stained with anti-Gr-1 are shown (A). Myeloperoxidase (MPO) activity at 6 h after ischemia/reperfusion (I/R) (B). Relative rate of accumulation of leukocytes and neutrophils during I/R (C). Representative FACS data, in which anti-CD45 or the isotype control was used to stain cells isolated from the sham, I/R, or I/R+F groups 12 h after I/R (D). Quantitative measurements of CD45+ cell number in the sham, I/R plus vehicle (I/R+V), and I/R plus fasudil (I/R+F) groups (E). Representative FACS data for CD45+ cells purified using immunobeads and stained with anti-Gr-1+ (F). Summary data of FACS analysis. The results of quantitative analysis of the number of Gr-1+ cells among CD45+ cells are shown (G). Data represent means from at least 4 mice each. * p<0.05, ** p<0.01. Fasudil attenuates chemotaxis and cytokine production of leukocytes in vitro. Chemotaxis and production of IL-6 were significantly reduced in the presence of fasudil (10μM) (H and I).

To examine the influence of fasudil on leukocyte recruitment to the ischemic myocardium, we assessed neutrophil density using immunofluorescence with the anti-Gr-1 antibody. In the I/R+V group, I/R injury resulted in significant neutrophil infiltration (Figure 3A). Gr-1+ cells were reduced in the I/R+F group compared with that in the I/R+V group. Each neutrophil contains a certain amount of myeloperoxidase (MPO). MPO activity in the heart reflects neutrophil infiltration. MPO activity in the sham-operated mice was relatively lower, whereas that in the I/R+V group was significantly increased (Figure 3B). I/R-induced increases in MPO activity were significantly lower in the I/R+F group than in the I/R+V group. Flow cytometric analysis confirmed that accumulations of leukocytes are significantly induced during I/R injury (Figure 3C). Twelve hours after I/R, the numbers of CD45+ and Gr-1+ cells in the heart were significantly increased compared to sham-operated mice. I/R-induced infiltration of CD45+ and Gr-1+ cells was significantly reduced in the I/R+F groups (Figure 3 D, E, F and G). These results suggest that fasudil administration attenuated leukocyte infiltration to the heart following I/R injury. We also examined the role of rho-kinase inhibition in mediating chemotaxis and cytokine production in leukocytes. Fasudil significantly attenuated fetal calf serum (FCS)-induced chemotaxis and concentration of IL-6 in condition media obtained from cultured leukocytes (Figure 3H and I), suggesting that rho-kinase inhibition have an essential role in mediating chemotaxis and cytokine production at the leukocyte level.

### Rho-kinase inhibition during I/R reduces myocardial infarct size through leukocytes activation

**Figure 4:**
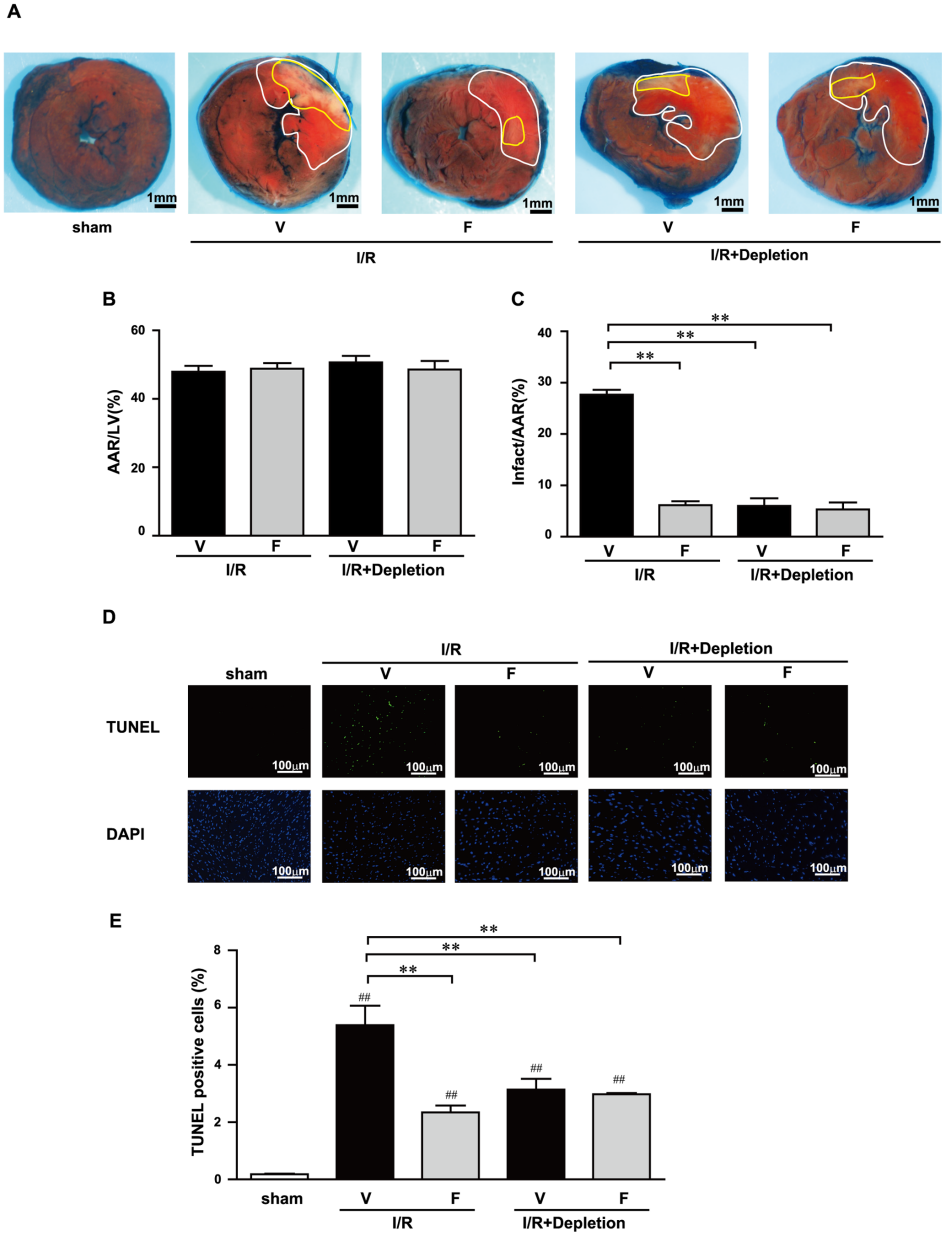
Fasudil reduces myocardial infarct size and apoptosis after I/R through leukocyte infiltration. Representative examples of myocardial infarction stained with Evans blue (EB) and triphenyl tetrazolium chloride (TTC) 24 h after reperfusion (A). Infarct area is marked with yellow lines. Risk area is marked with white line. The area at risk (AAR) was expressed as a percentage of the left ventricle (LV) (B). Myocardial infarct size expressed as a percentage of AAR (C). Representative photographs of terminal deoxynucleotidyl transferase dUTP nick-end labeling (TUNEL) staining in heart tissues obtained from mice that were subjected to 30-min ischemia and 24-h reperfusion and treated with vehicle (V) or fasudil (F), neutrophil depletion, or neutrophil depletion + fasudil. Fasudil reduces the I/R-induced increase in the number of TUNEL-positive myocytes (D). Quantitative measurement of the percentage of apoptotic myocytes (E). Data represent means from at least 5 mice each. ** p<0.01, compared with I/R+V; ## p<0.01, compared with the sham-operated group.

We examined whether inhibition of Rho-kinase was protective against I/R injury. The mean area at risk (AAR) in the left ventricle (LV) area was similar in the I/R+V and I/R+F groups (Figure 4B). I/R significantly increased infarct area (IA)/AAR at 24 h after reperfusion compared with the sham-operated mice. Administration of fasudil significantly attenuated the I/R-induced increase in IA/AAR (Figure 4A and C). These results suggest that Rho-kinase inhibition attenuates I/R-induced myocardial infarct size. To determine whether leukocytes mediate the protective effects of fasudil in I/R injury, mice were subjected to neutrophil depletion (Depletion) using anti-polymorphonuclear cell serum. The I/R-induced increase in IA/AAR were significantly reduced in the I/R+Depletion group (Figure 4 A, B and C). The administration of fasudil did not further decrease I/R-induced infarct size in the I/R+Depletion groups (Figure 4 A and C). These results suggest that the impact of Rho-kinase activation on the reduction of infarct size after I/R is mainly mediated by leukocyte infiltration, which is inhibited by fasudil.

I/R injury is associated with increased apoptosis in cardiac myocytes. To determine the extent of apoptosis, TUNEL-positive nuclei were counted among 5,000 nuclei in the AAR of each mouse. The numbers of TUNEL-positive myocytes were determined in the I/R+V, I/R+F, I/R+Depletion, and I/R+Depletion+F groups (Figure 4D). I/R-induced TUNEL-positive myocytes in the I/R+F, I/R+Depletion, and I/R+Depletion+F groups were significantly lower than those in the I/R+V group (2.3%±0.2, 3.1%±0.3, 2.9%±0.1 versus 5.4%±0.7, respectively, P<0.05; Figure 4E). These results suggest that fasudil inhibits I/R-induced apoptosis in cardiac myocytes.

### Rho-kinase inhibition reduces I/R-induced proinflammatory cytokine expression in the heart

**Figure 5:**
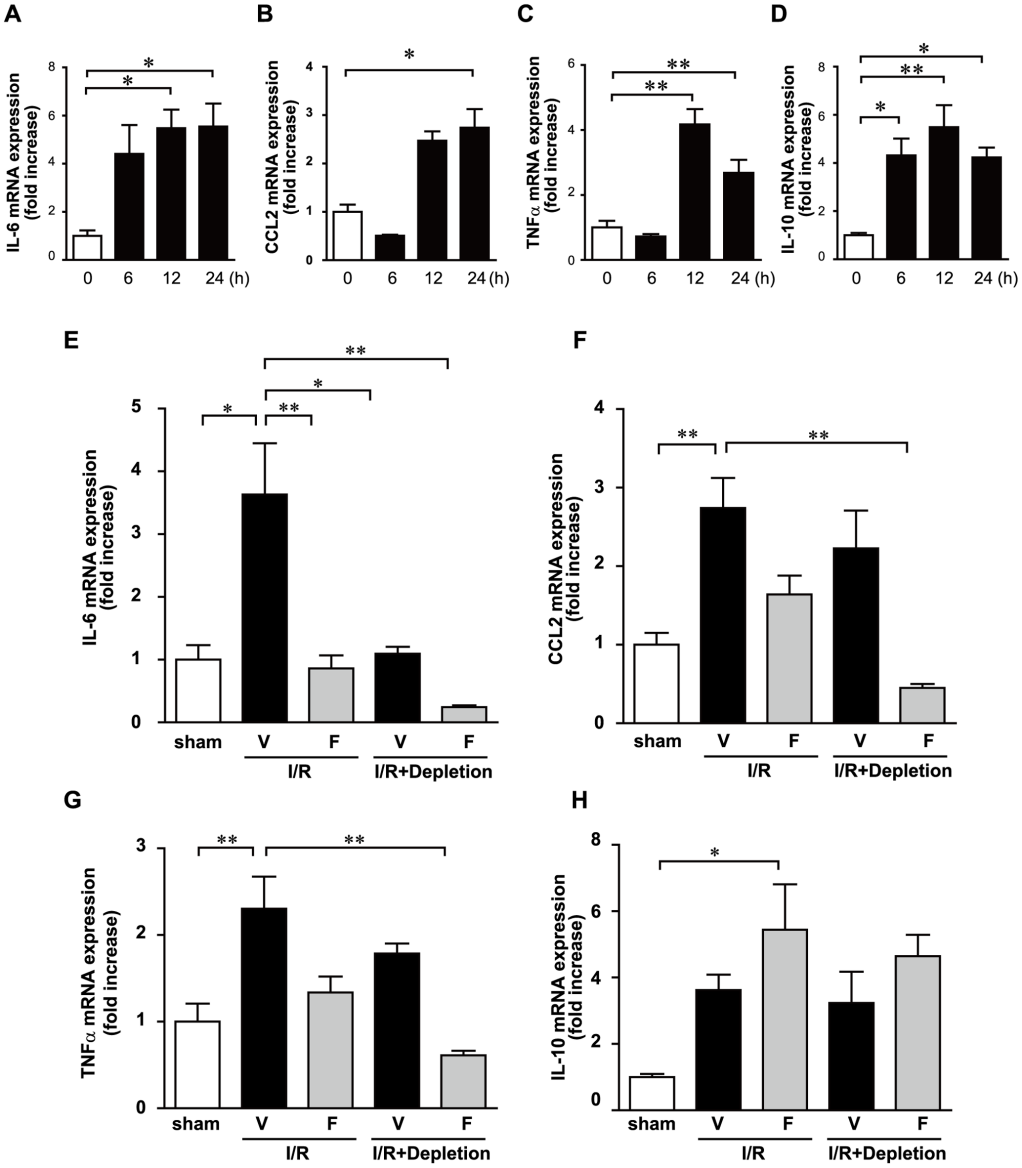
Fasudil reduces the I/R-induced inflammatory status in hearts partially through its action on leukocytes. I/R significantly induced expression levels of IL-6 mRNA (A), CCL2 mRNA (B), TNF-α mRNA (C), and IL-10 (D) in the heart. Data represent means from at least 4 mice each. * p<0.05, ** p<0.01. Fasudil and neutrophil depletion influence the expression of various cytokines 24 h after I/R in the heart. Expression levels of IL-6 mRNA (E), CCL2 mRNA (F), TNF-α mRNA (G), and IL-10 (H) in the heart. Data represent means from at least 5 mice each. * p<0.05, ** p<0.01.

Cytokine production is involved in the development of tissue injury after I/R. qRT-PCR analysis demonstrated that the mRNA expression levels of IL-6, CCL2, and TNF-α in the heart were significantly induced during I/R injury (Figure 5 A, B, C and D). Upregulation of IL-6 expression was significantly inhibited in the I/R+F group (Figure 5E). I/R-induced increases in CCL2 and TNF-α mRNA expression levels were not observed in the I/R+F group (Figure 5F and 5G). The anti-inflammatory cytokine IL-10 was upregulated in the I/R+F group (Figure 5H). Furthermore, administration of fasudil in the I/R+Depletion group significantly reduced the I/R-induced upregulation of IL-6, CCL2, and TNF-α expression. These results suggest that fasudil inhibits I/R-induced proinflammatory cytokine production in the heart from both leukocytes and cardiomyocytes.

## Discussion

The present study examined the effect of Rho-kinase inhibition on I/R injury in the mouse heart. We had several major findings: Rho/Rho-kinase pathways are activated in leukocytes after I/R in mouse models. Fasudil, a Rho-kinase inhibitor, prevents I/R-induced cytokine production in leukocytes and leukocyte infiltration to the heart after I/R. Administration of fasudil and depletion of neutrophils reduce I/R-induced infarct size and myocyte apoptosis in the heart. In this study, the administration of fasudil along with depletion of neutrophils resulted in no additional reduction of I/R-induced infarct size. These results suggest that the fasudil-induced reduction in infarct size after I/R is mainly through the inhibition of Rho-kinase activation in leukocytes.

Inflammatory reactions such as the accumulation of inflammatory cells and proinflammatory cytokines have been implicated in reperfusion injury [25]. Proinflammatory cytokines promote further inflammatory cell adhesion and infiltration into the myocardium and enhance acute tissue injury. Neutrophils are a major source of cytokines in the heart after I/R [11]. It has been reported that Rho-kinase regulates cytokine production in leukocytes in mouse models of severe inflammation [26]. In this study, we demonstrated the I/R-induced expression of proinflammatory cytokines, including IL-6, CCL2, and TNF-α in both serum and leukocytes. Rho-kinase inhibition reduced these cytokine increases. ACS is sometimes accompanied by systemic changes in inflammation, coagulation, metabolism, and gene expression in the liver [20], [27], [28]. Gene expression profiles of leukocytes in ACS patients were quite different from those in control patients [29]. We confirmed the activation of the Rho-kinase pathway in mouse leukocytes after I/R (Fig 1). Taken together, these results suggest that I/R injury cause inflammatory cytokine expression in leukocytes through Rho-kinase activation. This idea is supported by the observation that Rho-kinase activity in peripheral blood leukocytes is associated with coronary artery disease [15], [30] and acute ischemic stroke [31].

An increase in the peripheral leukocyte count is associated with the outcome of acute myocardial infarction. Neutrophils contribute to reperfusion injury by obstructing capillary vessels, producing vasospastic substances, and releasing inflammatory cytokines[11]. Our data and several experimental studies have indicated the efficacy of neutrophil-depleted reperfusion because neutrophils and their products play a significant role in reperfusion injury [32]. After open-heart surgery, neutrophil-filtered blood reperfusion significantly reduced clinical and biochemical markers of myocardial reperfusion injury. The cardioprotective effects of Rho-kinase inhibition may be related to regulation of neutrophil activity, considering that fasudil administration significantly reduced neutrophil infiltration in the ischemic myocardium (Figure 3). Furthermore, Bao et al. reported that the protective effect of Rho-kinase inhibition was not observed in isolated perfused rat hearts [14]. We confirmed that the depletion of neutrophils significantly reduced I/R-induced myocardial infarct size and this effect was equal to that of fasudil administration with depletion. These observations suggest that Rho-kinase pathway inhibition plays a pivotal role in the recruitment of leukocytes and reduction of infarct size *in vivo*.

The cardioprotective effects of the pharmacological inhibition of Rho-kinase during I/R, which included reduced infarct size and cardiomyocyte apoptosis in our study, has been experimentally verified. Y-27632, another Rho-kinase inhibitor, reduced infarct size after I/R and cardiomyocyte apoptosis by attenuating I/R-induced inflammatory responses and the cytokine expression level is increased [14]. Myocardial apoptosis is initiated shortly after ischemia, is enhanced by reperfusion, and contributes in part to myocardial death [33]. The number of TUNEL-positive cells was lower after fasudil treatment in our study. We also demonstrated that fasudil inhibited I/R-induced inflammatory cytokine expression in the heart. I/R induced the expression of proinflammatory cytokines including IL-6, CCL2, and TNF-α in the hearts. Rho-kinase inhibition reduced the I/R-induced upregulation of IL-6 expression levels but not those of CCL2 and TNF-α. Depletion of neutrophils tends to reduce the I/R-induced increases of these cytokines. Interestingly, the administration of fasudil with depletion of neutrophils significantly reduced the I/R-induced cytokine expression levels of IL-6, CCL2, and TNF-α in the heart. The action of fasudil, which inhibits leukocyte infiltration, partially decreases cytokine production in the heart. Inhibition of the Rho/Rho-kinase axis by fasudil in cardiac myocytes also contributes to the reduction of cytokine production in the heart. Okamoto et al. reported that Rho-kinase activation in cardiomyocytes has an important role in cardiac remodeling during pressure overload[34]. These effects may lead to the inhibition of stress-induced inflammatory responses and diminished cardiac remodeling following I/R injury.

Based on these results, we suggest that reduced cytokine expression may play an important key role in the cardioprotective effect of fasudil. Here, we also demonstrated that fasudil administration increased the expression of IL-10, an anti-inflammatory cytokine [35], [36]. Proinflammatory mediator downregulation is a mechanism of reducing I/R injury, whereas IL-10 upregulation may be another mechanism that contributes to the anti-inflammatory and anti-apoptotic effects resulting from fasudil administration. IL-10 has been reported to increase in patients and animal models following I/R [37], [38], and this reduces proinflammatory cytokine production and inhibits TNF-α-induced oxidative stress and apoptosis in cardiac myocytes [10]. These observations suggest that the cardioprotective effects of fasudil are ascribed not only to the suppression of proinflammatory cytokine expression, but also to the increase in the expression of anti-inflammatory cytokines following I/R.

In conclusion, the present study demonstrates that fasudil, a Rho-kinase inhibitor, reduces I/R injury by inhibiting cardiomyocyte apoptosis, inflammatory cytokine production, and leukocyte accumulation to the heart. These results suggest that the Rho-kinase pathway plays a pivotal role in not only the heart but also leukocytes during I/R, and that Rho-kinase inhibition may be an additional therapeutic intervention for the treatment of ACS.
